# Cylindrical Crystallization of Ca^2+^-ATPase and Its Potential Role in Sarcoplasmic Reticulum Dynamics

**DOI:** 10.3390/ijms27104314

**Published:** 2026-05-12

**Authors:** Jun Nakamura, Genichi Tajima, Makiko Suwa, Chikara Sato

**Affiliations:** 1 Jun Nakamura Institute, Moniwa-dai 2-3-21, Taihaku-ku, Sendai-shi, Miyagi 982-0252, Japan; 2 Institute for Excellence in Higher Education, Tohoku University, 41 Kawauchi, Aoba-ku, Sendai-shi, Miyagi 980-8576, Japan; 3 Biological Science Course, Graduate School of Science and Engineering, Aoyama Gakuin University, 5-10-1 Fuchinobe, Chuou-ku, Sagamihara-shi, Kanagawa 252-5258, Japan; 4 School of Integrative and Global Majors (SIGMA), University of Tsukuba, 1-1-1 Tennodai, Tsukuba-shi, Ibaraki 305-8577, Japan; 5 Division of Immune Homeostasis, Department of Pathology and Microbiology, Nihon University School of Medicine, 30-1 Oyaguchi-Kamimachi, Itabashi-ku, Tokyo 173-8610, Japan; 6 Division of Microbiology, Department of Pathology and Microbiology, Nihon University School of Medicine, 30-1 Oyaguchi-Kamimachi, Itabashi-ku, Tokyo 173-8610, Japan

**Keywords:** ryanodine receptor, calcium trafficking, muscle contraction, protein crystallization, organelle, excitation-contraction coupling, skeletal muscle

## Abstract

How do ryanodine receptors (RyRs) open simultaneously to trigger the contraction of whole myofibrils within a large skeletal muscle cell? One possible answer is the uniformity of mechanosensitive RyRs, which is mechanically forced by the neighboring environment, including proteins. Here, we review papers addressing this proposed “mechanical sarcoplasmic reticulum (SR) paradigm”. Crystals of the molecular complexes comprising RyR and L-type voltage-gated Ca^2+^ channels were observed at the T-tubule/SR junction in situ using cryo-electron tomography. Observations of the SR vesicles isolated from rabbit and scallop cross-striated muscles using negative staining and transmission electron microscope raised a hypothesis of dynamic rearrangement of the Ca^2+^-ATPase (ATPase) molecules in response to cytoplasmic calcium concentration, as follows: (i) At a low calcium concentration where the ratio of operating ATPase molecules to the total molecules is at a submaximal level, the ATPase molecules form, at least in part, their cylindrical crystals in the SR membrane with the help of ATP; this results in the elongation of the SR vesicles. (ii) High concentrations of calcium, at which the ratio of operating ATPase molecules is maximal, reversibly collapse the ATPase crystals to transform the elongated vesicles into round forms comprising tightly attached crystal patches. These data further lead to the idea that the reversible growth of cylindrical ATPase crystals provides a dynamic crystalline network, which acts as an “SR membrane-endoskeletal motor” to manipulate the SR movement. The possibility of interactions between ATPase crystals and neighboring RyR crystals is also discussed.

## 1. Introduction

The sarcoplasmic reticulum (SR), which extends throughout the entire muscle cell, plays a central role in regulating cytoplasmic calcium concentration in skeletal muscle cells [1,2,3,4]. Following the synchronized release of calcium ions from the SR through ryanodine receptor channels (RyRs) to initiate muscle contraction [5,6], the Ca^2+^-ATPase (ATPase) of the SR facilitates muscle relaxation by pumping the released calcium ion back into the SR lumen, a process driven by ATP hydrolysis [1,2,3,4]. Why are RyRs able to open simultaneously to trigger the contraction of whole myofibrils within a long skeletal muscle cell? One possible answer is the uniformity of RyRs, which is mechanically forced by the neighboring microenvironment, including proteins and lipids within the SR. Here, we review papers that can address this proposed “mechanical SR” paradigm.

Various methodologies and techniques have been used to study SR proteins. The three-dimensional (3D) structures of RyR1 tetramer have been investigated in vitro, and its structure-function relationships have been precisely analyzed using cryo-transmission electron microscopy (cryo-TEM) reconstruction in combination with other methods [7,8,9,10,11,12,13,14,15,16,17,18,19,20,21,22,23,24]. Different structures have been captured under various conditions, including closed-state structures. Some open-state structures were captured with the assistance of exogenous pharmacological activators, such as caffeine, ryanodine, PCB-95, and diamide [10,12,15] in different conditions and in a lipidic environment [20]. Primed-state structures were captured in different conditions [12]. Structure-function relationships have been further studied in combination with regulatory proteins and factors, including calmodulin [14,16], FKBP12 [8,12], nucleotide derivatives [25], S100A1 [23] and statin [24].

These structures suggest dynamic movements of RyR1 tetramer during gating, which involves global conformational changes in the cytoplasmic assembly, accompanied by local changes in the transmembrane domain. This process includes the bending of the S6 transmembrane segment and consequent pore dilation [7,8,9,10,12,18,26], and the movement is allosterically regulated by modulation factors [8,10,12,18,23,26]. The RyR1 tetramer is believed to change its overall height and diameter during the open-to-close transition.

Furthermore, Wagenknecht et al. clearly visualized RyR1 crystallization in a two-row arrangement on the terminal cisternae (TC) of the SR at the triad junction in situ using cryo-electron tomography (cryo-ET) [25,26]. Similar crystallization has also been observed using traditional transmission electron microscopy (TEM) methods in both vertebrates [27] and invertebrates [28]. Moreover, Xu et al. revealed that the two-column ordered molecular crystal has an alternating checkerboard-like arrangement of L-type voltage-gated dihydropyridine receptor (DHPR)-RyR complexes and RyR-only complexes at the triad junction using cryo-ET combined with cryo-FIB milling [29]. Further subtomogram averaging indicated that these complexes include the regulator proteins FKBP12 and calmodulin. These observations strongly suggest a direct physical interaction between RyR1 and DHPR during excitation-contraction coupling (ECC) [30]. Finally, Franzini-Armstrong’s group clearly visualized in situ that the distance between individual DHPRs within the complex decreased in response to ryanodine-treatment of the RyRs using freeze fracture TEM, providing convincing evidence for the mechanical linkage between DHPRs and RyR1 [31].

The structures of RyR2 tetramer and its structure-function relationships have been studied [32,33] in relation to its modulators—including calmodulin [34], FKBP12.6, phosphorylation [35] and ARM210 [36]—as well as various mutations [37,38] using cryo-TEM reconstructions and other techniques. These studies precisely analyzed the role of RyR2 in Ca^2+^-induced Ca^2+^ release (CICR). Furthermore, a combination of line-scan confocal fluorescence microscopy, resin-embedding/staining electron tomography and dSTORM imaging revealed that the distribution and crystallization of RyR2 on cardiac myocytes are dynamically affected by phosphorylation as well as FKBP12 and FKBP12.6 binding [39].

The ATPase is a major protein in the SR, occupying about 90% of the proteins integrated in the longitudinal SR membrane [40,41]. The structure of the ATPase molecule and its dynamic movements within the molecule have been precisely studied in vitro using X-ray crystallography [42,43]. Regarding the arrangement of the ATPase molecules in SR vesicles isolated from rabbit cross-striated skeletal muscle, Scales and Inesi [44] first found an ordered disposition in the absence of ATP. They reported that the average particle density (particles/μm^2^) of the 40 Å domains of the monomer ATPase molecule on the SR surface is 3 to 4 times greater than that of the 85 Å particles observed using negatively stained TEM in the SR vesicles. These observations were followed by Saito et al. [45]. The findings suggest tetramer formation for ATPase molecules within the membrane. This is also consistent with the kinetic hypothesis [46,47,48,49,50] that an oligomeric interaction is involved in the calcium transport reaction.

However, other studies indicate that the ATPase molecules exhibit mostly disordered disposition and form random contacts within the membrane [51]. Is tetramer formation necessary for their Ca^2+^ pump activity? It appears not, because ATPase molecules that are successfully monomerized with a detergent retain their calcium-pumping function in the presence of ATP [52]. This is also supported by the fact that monomeric ATPase molecules reconstituted with an excess of phospholipids exhibited a high coupling ratio (1.0) between calcium transport and ATP hydrolysis [53]. Based on X-ray crystallography of the monomerized ATPase [54,55], it is revealed that the calcium transport reaction is coupled with substantial conformational changes within a single molecule at the atomic level; that is, the functionality of the ATPase can be accounted for by a monomeric mechanism.

On the other hand, in the absence of ATP, two calcium-transport sites on each detergent monomerized ATPase molecule showed negative cooperativity in the binding of two calcium ions [56]. This behavior contrasts with the non- and positive cooperative binding patterns observed in the two native membranous ATPase conformers [57]. Based on this heterogeneous calcium binding in the absence of ATP, it was hypothesized that the ATPase molecules exist as two conformational variants of the same amino acid sequence, present in a 1:1 ratio within the SR membrane [57,58,59]; a corresponding tetramer model was subsequently proposed for these variants [58].

Moreover, it has been suggested that an intermolecular interaction among the ATPase molecules enhances the “internalization (or occlusion)” of calcium, bound to the calcium transport site of the ATPase [60,61]; the internalization is thought to be an integral part of coupling enzymatic catalysis and calcium translocation [60]. These earlier observations about the ATPase oligomer functionality do not seem to conflict with the ATPase monomer functionality. Namely, they suggest that membranous ATPases take various functional conformations, as discussed earlier [62]; the monomeric ATPase can perform the fundamentals of the calcium transport function. It is therefore thought that the study of these monomeric and oligomeric types of the ATPase is complementary. Despite our persistent advocacy for an oligomeric model of the ATPase molecules, definitive structural evidence has not yet been obtained.

To bridge the gap between the monomer and oligomer models, we employed negatively stained TEM combined with a quick sample preparation method, analyzing the distribution of ATPase molecules on the cytoplasmic surface of SR vesicles isolated from rabbit cross-striated white muscle and scallop cross-striated adductor muscle in the presence of ATP [63,64,65]. Note that negative staining involves drying and staining processes that might produce artifacts. Earlier, Castellani et al. [66,67] indisputably showed the presence of similar cylindrical crystal arrays of scallop ATPase molecules, not only using negatively stained TEM of isolated scallop SR vesicles with and without ATP in vitro, but also using the freeze-fractured/deep-etched tissue of scallop whole striated muscles in situ. The observed cylindrically crystallized SR tubule with a fairly uniform diameter in situ is presumed to be a native longitudinal SR in the muscle [68]. It was also observed that ATP did not affect the ATPase crystallization in vitro [67]. However, for the collapse of the crystalline array in the isolated SR vesicles, 0.1 millimolar (mM) Ca^2+^ (beyond its physiological concentration within the muscle cell) was required [67]; thus, the physiological meaning of the crystallization remained not fully clarified.

Recently, we observed the dynamic movements of rabbit and scallop SRs and their ATPase molecules under physiological conditions [63,64,65]. In this review, we outline our observations and those of other groups concerning calcium regulation in muscle contraction and relaxation. Given the lack of experimental support, the discussion remains somewhat speculative.

## 2. ATP-Induced Crystallization of ATPase Molecules

### 2.1. In the Case of Rabbit SR

We previously examined the effects of various nucleoside triphosphates (ATP, CTP, GTP, ITP, and UTP) on the binding of two calcium ions to the calcium transport sites of rabbit ATPase molecules, both before and after their solubilization with detergent [69,70]. The results showed that: (i) ATP enhances calcium-binding performance (both affinity for calcium and the cooperativity of the two calcium transport sites in binding two calcium ions), regardless of the disruption of the ATPase organization by detergent. (ii) The other NTPs degrade the calcium affinity of the detergent-solubilized ATPase molecules; their effect on the cooperativity of two calcium binding sites was not experimentally determined.

**Figure 1:**
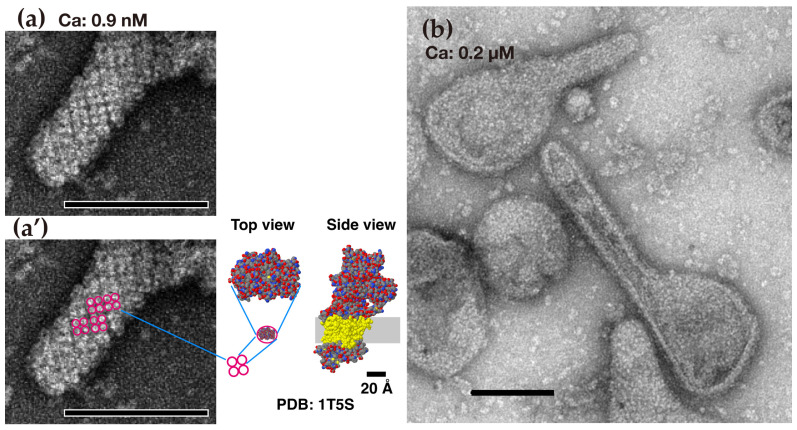
Typical images of tightly elongated rabbit SR vesicles, observed in the presence of ATP. (**a**) Vesicle with an ATPase crystal array at 0.04 nanomolar (nM) Ca^2+^. (**a’**) clear tetramer units of the ATPase particles shown in (**a**) are marked with red circles. (**b**) Vesicles without ATPase crystal array at 0.2 micromolar (µM) Ca^2+^. The illustrations between (**a’**,**b**) show the atomic models (top and side views) of a rabbit ATPase monomer (PDB 1T5S) [72] in the *E*_1_ state with bound calcium, prepared with Jmol (https://launchpad.net/jmol). Scale bars in (**a**,**a’**,**b**): 100 nm (Reproduced from [63]).

These observations led to the ideas that (i) the oligomerization of ATPase molecules integrated in the membrane protects them from interference by the other NTPs within the muscle cell, and (ii) the conformation of the ATPase molecules is transformed into a fully matured form with the assistance of the ATP, independent of their state (membrane-bound/oligomeric or solubilized/monomeric). This concept of ATP-supported ATPase transformation may be supported by the recent proposal that ATP acts as a biological hydrotrope [71]; it was reported that ATP (5–10 mM) inhibits the heat-induced aggregation of egg white proteins in a dose-dependent manner. Therefore, the disposition of ATPase in the rabbit SR membrane was recently studied in the presence of ATP [63]; it is likely that such an effect of ATP on this disposition had not previously been examined. Subsequently, an ATP-supported and calcium-sensitive orderly disposition of the ATPase molecules was discovered, as described below. To prepare TEM samples of the SR vesicles, the vesicles were incubated overnight with 23 mM ATP at 0 °C to stabilize the disposition of the ATPase molecules [63]. The following observations were made (see Figure 1).

(i) At a low concentration of calcium (<0.9 nM), where the ATPase molecules scarcely transport Ca^2+^ in the presence of ATP, many vesicles exhibited a protrusion composed of cylindrically crystallized ATPase tetramers. These results suggest that some of the round SR vesicles, which showed no orderly molecular disposition on their surface in the absence of ATP, were transformed into tightly elongated vesicles with a crystallized tetramer-array, although further agglomeration between the isolated SR vesicles also occurred.
(ii) As the calcium concentration increases to 0.2 µM—the concentration at which the ATPase molecules fully perform their transport activities—the ratio of elongated vesicles with crystallized tetramers to total vesicles decreases. These results suggest that the crystalline array in the elongated vesicles gradually becomes disordered as the [Ca^2+^] increases.

Considering the facts that (i) rabbit ATPase is the predominant protein component (~90%) [40,73] in the longitudinal SR tubules [41] and (ii) the transmembrane domains of ATPase molecules [74,75] form close, but disordered contacts with each other in the absence of ATP [51], the aforementioned correlation between tight elongation and the ordered ATPase array leads us to a hypothesis: ATPase molecules may form a dynamic calcium-sensitive, membrane-endoskeleton to elongate SR vesicles in vivo. Taking into account the observation of pentagonal vesicles with an ordered ATPase array under this condition [63], we infer that the SR not only elongates but also expands to contribute to the mesh stem when the ATPase molecules crystallize; the observed pentagonal vesicles [63] might be related to intermediate cisternae of the SR [76].

However, further examination of the effect of non-hydrolyzable ATP analogs on ATPase disposition is required to test the hypothesis of ATP-supported ATPase transformation. Here, alternative hypotheses regarding the ATPase disposition, different from the ATP-supported model, are not excluded, such as those based on the functionality of the ATPase monomer [52,53,54,55], lipid-driven remodeling [77,78,79], and membrane-remodeling proteins [80,81,82].

### 2.2. In the Case of Scallop SR

The scallop ATPase was the predominant protein component (~85%) in the SR vesicle preparation employed in the earlier study [66], similarly to that of the rabbit [40,73]. However, the observed ATPase crystallization had low calcium sensitivity [67], and the calcium transport activity was much lower (~4 nmol Ca^2+^/mg of protein/min) [66] than that of rabbit (800–6000 nmol Ca^2+^/mg of protein/min) [40,83]. Therefore, the physiological significance of the crystallization remained not fully understood [84].

In this study, we employed an isolated scallop SR sample with high calcium transport activity [83], which is comparable to that of rabbit SR preparations [40,83], although the content of the ATPase protein in the scallop SR proteins was somewhat lower (40–50%). We consider the use of this SR sample to be important for further studies. The preparation method for the isolated vesicles was basically the same as that described by Ogawa [85]. The key feature of this method is the use of NaHCO_2_/HCl (Sodium bicarbonate/hydrochloric acid) instead of amine compound buffers to adjust the pH of the homogenate during the homogenization of the scallop muscle. Our additional points to note in preparing scallops for SR sampling are as follows: (i) Fresh seawater was stored for more than a few months at room temperature to stabilize its microbiome, after which it was used for scallop culture. (ii) Active scallops were selected after cultivating them at 4 °C overnight in fresh, unused seawater.

Among the SR vesicles employed, the protrusion of each vesicle tended to comprise a homogenous unit. Based on this unit, the arrays in all vesicles were roughly classified into three types, which are mainly composed of “tetragon”, “two-rail” and “monomer” as well as “crystal patches” of the scallop ATPase.

Thapsigargin, a specific inhibitor of rabbit ATPase [86,87,88], inhibited the crystallization of the scallop ATPase molecules. The ATP-supported stabilization of crystalline arrays may be described based on the biological hydrotrope model of ATP [71], similarly to the case of rabbit ATPase crystals [63]. To verify this hydrotrope model, the effect of ATP on ATPase crystallization should be further investigated. The secondary structure of the scallop ATPase molecule is proposed to contain ten transmembrane and five ‘stalk’ domains, along with two large cytoplasmic regions [89], resembling that of the rabbit ATPase [74,75]. Considering that (I) scallop ATPase is the predominant protein component (~85%) [66] in isolated longitudinal SR vesicles, and (II) earlier negative-staining electron microscopic studies of the scallop molecule have revealed a large cytoplasmic region [90], the results for the scallop molecule appear comparable to those of the rabbit molecule (Figure 1) [72]. Therefore, Scallop ATPase molecules might also act as a membrane endoskeleton to elongate the SR, as proposed for rabbit ATPase [63].

**Figure 2:**
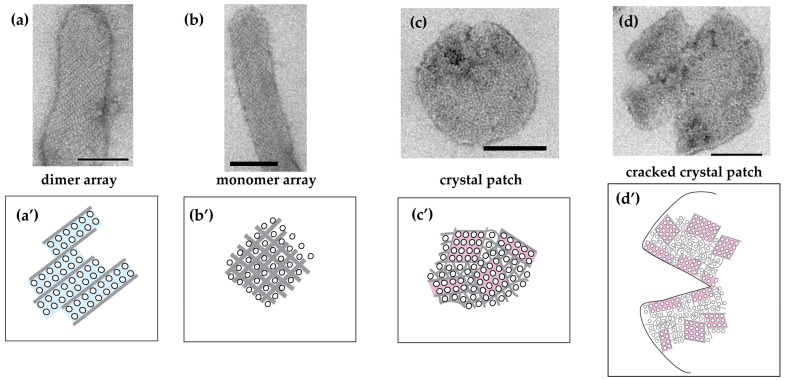
Typical images of the elongated SR vesicles isolated from scallop muscle, exhibiting crystalline dispositions and crystal-patch assembly of ATPase molecules in the presence of ATP. (**a**) A tightly elongated vesicle with an ATPase dimer array. (**b**) A tightly elongated vesicle with an ATPase monomer array. (**c**) A round vesicle with an ATPase crystal-patch assembly. (**d**) A cracked vesicle with an ATPase crystal-patch assembly. (**a’**–**d’**) Schematic illustrations corresponding to the four types of ATPase arrays in the vesicles shown in panels (**a**–**d**). Scale bars in (**a**–**d**): 100 nm (Reproduced from [65]).

Regarding scallop SR vesicles with ATP, the calcium-dependence of their vesicular shapes and ATPase dispositions in vitro [65] can be summarized as follows:

(i) At a low calcium concentration (≤1.4 µM), tightly elongated forms containing ordered arrays of two-rail and/or monomer units were observed in 4.2% of the vesicles (average appearance rate; SD = 2.5, *n* = 16) (Figure 2a,a’,b,b’). As the calcium concentration increased, the proportion of observed elongated vesicles decreased to 0.1% (SD = 0.2, *n* = 6), and they almost completely disappeared at ≥18.0 µM [Ca^2+^].
(ii) Round SR vesicles (Figure 2c,c’) were dominant over the entire range of calcium concentrations examined. However, their appearance rates can be roughly classified into two groups depending on the calcium concentration: a moderately high rate of 90.2% (SD = 6.8, *n* = 16) at ≤1.4 µM [Ca^2+^] and a very high rate of 99.1% (SD = 0.3, *n* = 6) at ≥18.0 µM [Ca^2+^].
(iii) At a high calcium concentration (≥18.0 µM), ATPase dispositions on round vesicles primarily formed tightly attached crystal patches.
(iv) The ATPase crystallization was rapidly (<1 min) reversible upon increasing or decreasing the calcium concentration.

Based on observations of ATPase dispositions under different [Ca^2+^] levels, with and without [ATP] (5 mM) [64,65], we have hypothesized the following:

(i) In round vesicles stored at a high [Ca^2+^] (>180 μM) without ATP, the crystal patch assemblies of ATPase molecules begin to transform into assemblies of ~100 Å square tetragons after incubation at a low [Ca^2+^] (≤1.3 μM), subsequently developing into cylindrical “tetragon” arrays.
(ii) At the low [Ca^2+^] without ATP, these “tetragon” arrays are unstable.
(iii) With the addition of ATP, these arrays transform into more stable, cylindrical “two-rail” arrays of high regularity.

During observations, cracks occurred on some of the ATP-treated round scallop vesicles (Figure 2d,d’), exhibiting the following characteristics.

(i) Cracks were frequently observed at the edges of the round SR vesicles.
(ii) Each cracked vesicle was covered with a very tight assembly of small ATPase crystal patches (<ca. 20–40 nm squares).

The crack might be created during the drying process of negative staining, attributable to a large surface tension, as suggested by time-lapse monitoring of liquid-phase electron microscopy (ASEM (atmospheric scanning EM)) of deformation of palladium-based nanowires in a liquid drop [91] and drying mechanics of MEMS (Micro Electro Mechanical Systems) [92,93,94,95]. These observations, along with the fact that no distinct crack was observed in the absence of ATP, suggest structural differences between the SR vesicles with and without ATP. One hypothesis is that the stable ATPase crystal patches gather to form a tight assembly with the help of ATP, causing the vesicle to transform into a rigid 3D sphere. Subsequently, the dehydration-induced surface tension during negative staining might cause the crack on these rigidified vesicles. It is noted that such a vesicle crack was also observed in the rabbit SR vesicles treated with ATP, although this was not mentioned in our earlier paper [63], due to insufficient understanding of the phenomena at that time.

## 3. A Possible Dynamic Role of SR in the Regulation of Muscle Contraction

### 3.1. A Calcium-Dependent and Autonomous Elongation-Contraction Model

**Figure 3:**
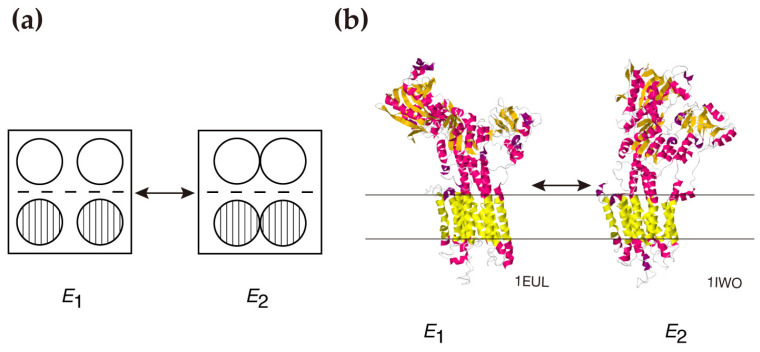
Schematic representation of the dynamic movement models of the rabbit ATPase molecule(s) accompanying *E*_1_–*E*_2_ transition. (**a**) Intermolecular movement of the molecules on the cytoplasmic surface of the SR membrane. Open and hatched circles denote the two different types of molecules (Reproduced from Ref. [58]). (**b**) Intramolecular movement of the molecule inside and outside the SR membrane. Structural data were taken from PDB and PDBTM (*E*_1_, PDB ID: 1EUL [42]; *E*_2_, PDB ID: 1IWO [43], and the *E*_1_ and *E*_2_ ribbon models were prepared with Jmol.

Regarding the calcium transport-coupled movement of ATPase molecule(s)—accompanied by the *E*_1_ (high affinity state for calcium) to *E*_2_ (low affinity state for calcium) transition—two models and related experimental data have been reported for rabbit SR: a model of monomer-dimer transition of the ATPase molecules (Figure 3a) [58,96] and observations of dynamic, intramolecular movement of the molecule (Figure 3b) [42,43]. Taking these earlier findings into account, it is hypothesized that the calcium sensitivity of the orderly ATPase disposition in scallop SR is indirectly produced by calcium transport-coupled structural movement between and within the ATPase molecules.

**Figure 4:**
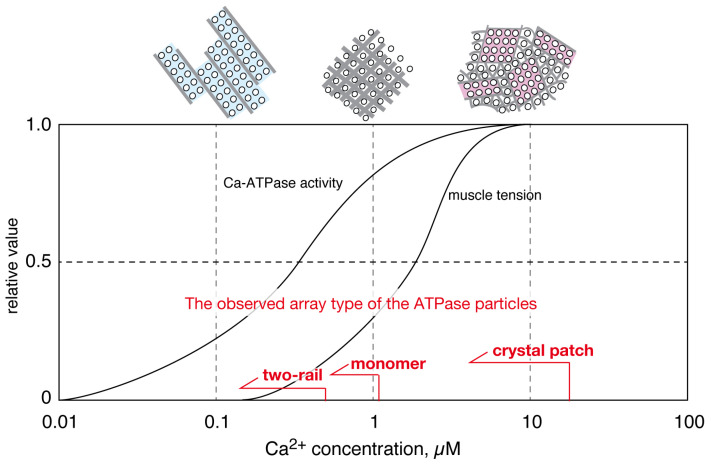
Schematic representation of calcium dependencies of ATPase activity [83], striated muscle tension development [98] and ATPase disposition of scallop in the presence of ATP. The calcium dependence of tension development was examined using a caged calcium molecule and a chemically skinned fiber bundle of the striated adductor muscle, and the output of the tension transducer was recorded on a pen recorder and as a filtered signal on a Nicolet ‘Explorer’ digital oscilloscope [98].

The ratio of operating ATPase molecules to the total number of active ATPase molecules was estimated from the calcium-dependent profile of Ca^2+^-ATPase activity. At Ca^2+^ concentrations of 0.01, 0.6, 1.4, and 10–20 µM, the ratios were estimated to be approximately zero, 0.5, 0.8 and 1.0, respectively (Figure 4) [65] (see [97] for information on active ATPase molecules in rabbit SR and their content within total ATPase molecules, although no information is available for scallop molecules). Conversely, the tension development of the scallop striated muscle was estimated to be less than its half-maximal level below 1.4 µM Ca^2+^, reaching its maximal level at 10–20 µM Ca^2+^ [98].

These calcium-dependent profiles of ATPase activity and tension development imply that the calcium transport activity of the ATPase molecules almost reaches its maximal level at 10–20 µM Ca^2+^. At such high [Ca^2+^], cylindrical ATPase crystal arrays seem to be replaced with round assemblies of ATPase crystal-patches, as two-rail and monomer type arrays disappeared at Ca^2+^ concentrations of >0.6 and >1.4 µM, respectively. Furthermore, such a calcium-dependent transition of the disposition states was suggested for scallop SR vesicles even in the absence of ATP [64]. However, the observed ordered dispositions were found to degrade easily at a low concentration (~2 nM) of calcium, and the ratio of cylindrical vesicles to total vesicles was smaller; the details of their transition were not as precisely clarified as those for vesicles in the presence of ATP [65]. Taking these facts into account, the calcium transport-coupled movement of each ATPase molecule likely promotes the transition of the ATPase disposition under physiological ATP conditions.

On the other hand, Merton earlier proposed the presence of a refractory mode of muscle against electrical depolarization, on the basis of the contraction behavior of the adductor muscle of the human thumb after severe contraction (for ~2 min) [99]. It might reflect the presence of a specific mechanism, e.g., excitation-contraction coupling is interrupted at RyR1s because the RyR1s might be in a refractory mode due to possible differences in the environmental pressure exerted by the surrounding ATPase crystal. In this model, refractory mode might continue until the decrease in calcium concentration uniformly approaches a certain level in the cytoplasm.

In the case of vertebrate muscle, the calcium dependencies of tension development and SR ATPase activity have been obtained in vivo using skinned muscle cells [100] and in vitro using the membranous ATPase partially purified from the SR [69], respectively. Under physiological conditions with a millimolar concentration (5 mM) of ATP, the apparent calcium affinity (half-maximal concentration of calcium *(K*_0.5_)) for the ATPase activity was again markedly higher (~0.05 µM) than that (~0.5 µM) [100] for tension development. These data agree well with the physiological role of the SR in regulating contraction because a higher calcium affinity for ATPase activity than that for tension development is necessary to quickly remove calcium to relax the myofibril, although the calcium affinity for ATPase activity is only available in vitro. These results, mainly from the scallop SR vesicles, thus lead to the following hypothesis concerning the SR movement.

(i) At low calcium concentrations [Ca^2+^] ≤ 1.4 μM, where scallop ATPase activity is submaximal and muscle contraction is at or below half-maximum, ATPase molecules crystallize into a cylindrical network (including two-rail and monomer array types) to elongate the SR. This cylindrical structure is likely strong enough to resist the water surface tension encountered during the drying process of negative staining in TEM sample preparation. This suggests that these orderly arrays might generate a stretching pressure on nearby mechanosensitive RyRs, thereby possibly priming the RyRs for electrical depolarization (standby mode).
(ii) At high calcium concentrations ([Ca^2+^] = 10–20 µM), where both ATPase activity and muscle contraction are near maximum, the elongated cylindrical crystal actively shrinks, facilitated by the rearrangement and gathering of small ATPase crystal-patches.
(iii) The partial transformation of the SR into a round form might reduce the pressure exerted by the ATPase crystalline network on the RyR crystals. Instead, the weak surface tension formed by spherically attached ATPase crystal patches might pull the RyR molecules into a refractory mode.
(iv) The transition from two-rail arrays to monomer arrays, which is considered to occur between 0.6 and 1.4 µM Ca^2+^, could weaken the cylindrical crystal formation, leading to a decrease in the SR’s stretching force. This might induce partial closure of the RyR calcium channel.

The aforementioned discussion leads us to the following ideas.

**Figure 5:**
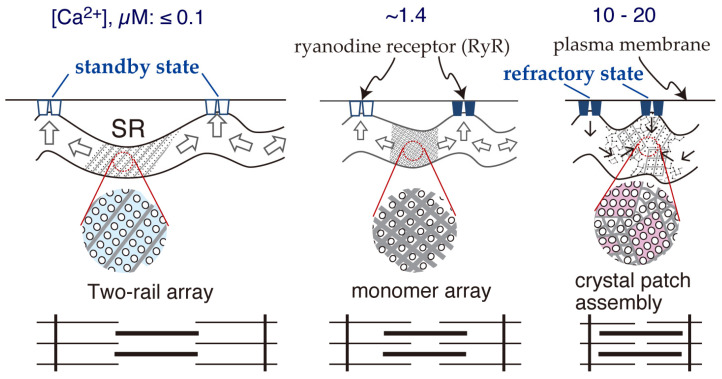
A model linking calcium-dependent crystallization of ATPase to contraction of the scallop muscle cell (see Figure 12 in Ref. [65] for details).

(i) The SR might function as a calcium-dependent, autonomous elongation-contraction machine, in addition to its primary role in muscle relaxation.
(ii) The reversible growth of the cylindrical ATPase array could provide an “ATPase membrane-endoskeletal motor”, which might manipulate SR movement as well as RyR activation, as illustrated in Figure 5.

The characteristics of the dynamic ATPase organization discussed here might be reflected in the low regularity of the observed ATPase crystallin array, as described in Section 2.2. Our hypothesis of ATPase crystallization-based morphological changes in the vesicles is expected to be strengthened by supporting evidence, for example, regarding the osmolarity effect using impermeant solutes and various buffer concentrations, as well as the effects of lipid composition.

### 3.2. The SR Network Could Act as a Bird’s-Eye View Monitor Surveying Calcium Concentration Within the Muscle Cell, Which Might Be Mechanically Transmitted to the RyRs

**Figure 6:**
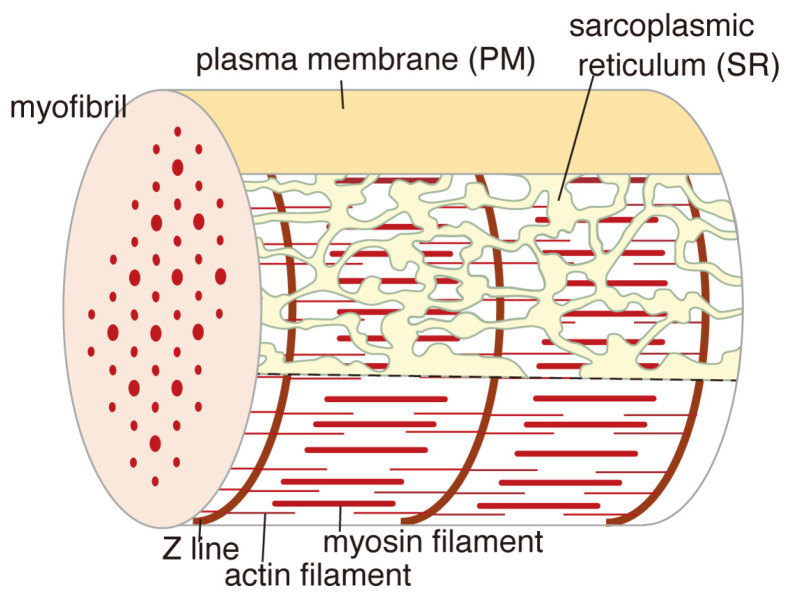
Diagram of a cross-striated adductor muscle cell in the resting state of a scallop (Reproduced from [64].)

Because each cross-striated adductor muscle cell of a scallop possesses a single SR network surrounding only one myofibril (Figure 6) [68], the entire SR network could monitor intracellular calcium information from a bird’s-eye view and might transmit this to the RyRs. Given that tubular SR elements extend beyond the Z line of myofibrils in vertebrates [76,101,102], a single SR network model has also been proposed for each vertebrate cross-striated skeletal muscle cell [76]. Therefore, the vertebrate SR might function as a calcium-dependent, autonomous elongation-contraction machinery, operated by the ATPase membrane-endoskeletal motor, which might mechanically influence RyRs’ crystals. This machinery is defined as the “Ca^2+^-ATPase membrane-endoskeleton model.”

Serum response factor (SRF) is a ubiquitously expressed transcription factor playing a vital role in contractility, cell migration, synaptic activity, inflammation and cell survival [103]. Patyal et al. generated a transgenic mouse model with cardiac-specific overexpression of SRF, which not only induces oxidative stress but also impairs calcium signaling through a drastic reduction in the expression of both Ca^2+^-ATPase and RyR2 in the isolated mouse cardiomyocytes [104]. This calcium signaling impairment is consistent with the mechanical coupling hypothesis between RyR crystals and ATPase crystals, suggesting potential mis-coupling between these crystals affected by the genetic modification.

While negatively stained TEM has been employed in investigations of ATPases and RyRs, this method includes the possibility of dehydration-induced deformation and staining artifacts [105]. However, negative staining is essential for sample optimization for cryo-TEM reconstruction due to its high reproducibility and throughput, and its ability to visualize membrane proteins with a molecular weight somewhat less than 140 kDa [106]. Therefore, the hypothesis proposed here should be further tested and confirmed using other in situ methods. The drying process can be avoided using cryo-TEM, which allows for the analysis of the structures and distribution/crystallization of very large protein complexes in situ [107]. Protein interactions have been studied using proximity ligation [108] and FRET [109]. Protein interactions in the SR and triad junctions should be further studied using these techniques and other methodologies. To verify this hypothesis, triad junctions of transgenic mice, especially those possessing ATPase without crystallization ability, are of great interest and should be studied in vivo and in situ using various techniques, including cryo-ET and optogenetics. Finally, to verify and further develop the discussed hypothesis, the participation of researchers from a wide range of fields is essential.

## 4. Conclusions

A hypothesis addressing a “mechanical SR” paradigm has been reviewed. The structures of RyR1 tetramer and ATPase molecule have been precisely investigated using cryo-TEM and X-ray crystallography, suggesting dynamic structural movements linked to their respective functions. Cryo-ET revealed the ordered crystalline disposition of RyR1/CaV1 complexes within the T-tubule/SR junction in situ. Based on observations of SR vesicles isolated from cross-striated muscles of two different species using negatively stained TEM, we propose a novel hypothesis for the regulation of skeletal muscle contraction, as follows. When cytoplasmic calcium concentration is low, and ATP is present, ATPase molecules reversibly form, at least in part, cylindrical crystals, leading to the elongation of the SR vesicles. Conversely, at higher calcium concentrations, these molecules assemble into spherical, attached crystal patches, resulting in the shortening of the vesicles. The stretching or contractive force generated by this reversible ATPase crystallization might be uniformly transmitted to push or pull neighboring mechanosensitive RyR crystals, thereby possibly regulating Ca^2+^ flow through the RyRs. Namely, SR might regulate calcium trafficking through mechanical interactions between ATPase crystals and RyR crystals, facilitating simultaneous contraction in a muscle cell.

## Data Availability

No new data were created or analyzed in this study. Data sharing is not applicable to this article.

## Abbreviations

The following abbreviations are used in this manuscript:

ATPase	Ca^2+^-ATPase
ASEM	Atmospheric scanning EM
cryo-TEM	cryo-transmission electron microscopy
EM	Electron microscope/microscopy
cryo-ET	cryo-electron tomography
MEMS	Microelectromechanical systems
RyR	Ryanodine receptor
SR	Sarcoplasmic reticulum
TEM	Transmission electron microscope/microscopy
TC	Terminal cisternae
SD	Standard deviation
3D	Three-dimensional
